# Missed Opportunities: Healthcare Encounters Prior to Sudden Unexpected Infant Death

**DOI:** 10.3389/fped.2022.880713

**Published:** 2022-05-03

**Authors:** Katherine O. Salada, Colleen M. Badke

**Affiliations:** ^1^Division of Hospital Based Medicine, Ann & Robert H. Lurie Children's Hospital of Chicago, Chicago, IL, United States; ^2^Department of Pediatrics, Northwestern University Feinberg School of Medicine, Chicago, IL, United States; ^3^Division of Critical Care Medicine, Ann & Robert H. Lurie Children's Hospital of Chicago, Chicago, IL, United States

**Keywords:** SUID (sudden unexpected infant death), SIDS (sudden infant death syndrome), safe sleep, infants, healthcare encounters

## Abstract

**Introduction:**

Sudden unexpected infant death (SUID) is the leading cause of death in children 28 days to 1 year of age. The study aim was to identify opportunities for healthcare professionals to provide families with education on sleep and prevention of SUID.

**Methods:**

We performed a retrospective chart review of SUID infants over 10 years (12/2010–12/2020). The study included patients 0–12 months who presented to single institution with SUID (including asphyxia, suffocation, and SIDS). Baseline descriptive characteristics, sleep patterns (location, position, co-sleeping, presence of pillows/blankets), and prior healthcare encounters (type, duration, frequency, timing) were described.

**Results:**

Thirty-five infants met inclusion criteria. Twenty-three percent of families routinely practiced unsafe sleep, while 63% practiced unsafe sleep at the time of SUID. All unsafe sleep behaviors increased during the SUID event compared to routine, including inappropriate location (60%), co-sleeping (46%), and inappropriate position (37%) at the time of SUID. There were 54 total healthcare encounters (mean 1.5 per patient +/− 2.1) prior to SUID. Primary care physicians (57%) and NICU (29%) were the most frequent prior healthcare encounters, however visits spanned multiple specialties. Twenty-six percent had a healthcare encounter within 7 days of their death.

**Discussion:**

We demonstrated the frequency and variability in healthcare encounters among SUID infants prior to their death. Majority of infants had prior healthcare encounters, with 26% seen by healthcare professionals within 7 days of their death. These results highlight the important role healthcare professionals across all specialties have the potential to play in educating families about safe sleep and SUID.

## Introduction

Sudden infant death syndrome (SIDS), a sub-category of sudden unexpected infant death (SUID), is the leading cause of death in infants 28 days to 1 year of age (1, 2). Although infant deaths from SUID are likely multifactorial, cases are often associated with unsafe sleep practices. Several factors have been identified as protective against SUID, including: supine position while sleeping, using a firm sleep surface, breastfeeding, offering a pacifier, room-sharing without bed-sharing, elimination of soft objects from the bed or under the infant, prevention of overheating, and avoidance of tobacco, alcohol, and illicit drugs (3–5). Given these associations, pediatricians recommend the pivotal aspects of infant safe sleep as the ABCs: Alone, on the Back, and in an empty Crib (6).

Healthcare professionals are uniquely positioned to educate new parents about infant safe sleep at every encounter. Safe sleep initiatives have historically targeted nursery care and neonatal intensive care unit (NICU) hospitalizations, with nursery-based safe sleep programs successfully resulting in reduced average SUID rates post-intervention (7). Studies addressing safe sleep during outpatient primary care physician visits, inpatient general pediatric admissions, and emergency room visits are limited, however promising new data shows that parent knowledge and practice of infant safe sleep are influenced by in-hospital interventions, with parents more likely to place their infants in a supine position and use a crib following intensive hospital educational efforts (8, 9). Studies have begun to look at how modeling safe sleep practices and educating parents in the hospital result in improved patient outcomes (8), yet this area is only beginning to evolve.

The 2016 American Academy of Pediatrics (AAP) Taskforce update on SIDS stated that healthcare providers should model safe sleep practices in the hospital (10). They further stress that providers should receive education on infant safe sleep and hospital policies should meet safe sleep standards (10). Prior to quality improvement efforts, only 0% to 32% of hospitalized infants slept in a safe environment (11–13). Improved adherence to safe sleep standards and modeling of safe sleep practices provide an opportunity to educate parents throughout their hospitalization. Although the historical approach of targeting nursery and NICU care for population-wide safe sleep initiatives remains important, directing SUID prevention initiatives toward the most frequent types of healthcare interactions may increase educational opportunities and better capture at-risk patients prior to their death. There is a paucity of published data on where and how infants interact with the healthcare system prior to SUID. Therefore, this study aimed to identify opportunities for healthcare professionals to provide families with education on safe sleep and prevention of SUID.

## Methods

**Figure 1:**
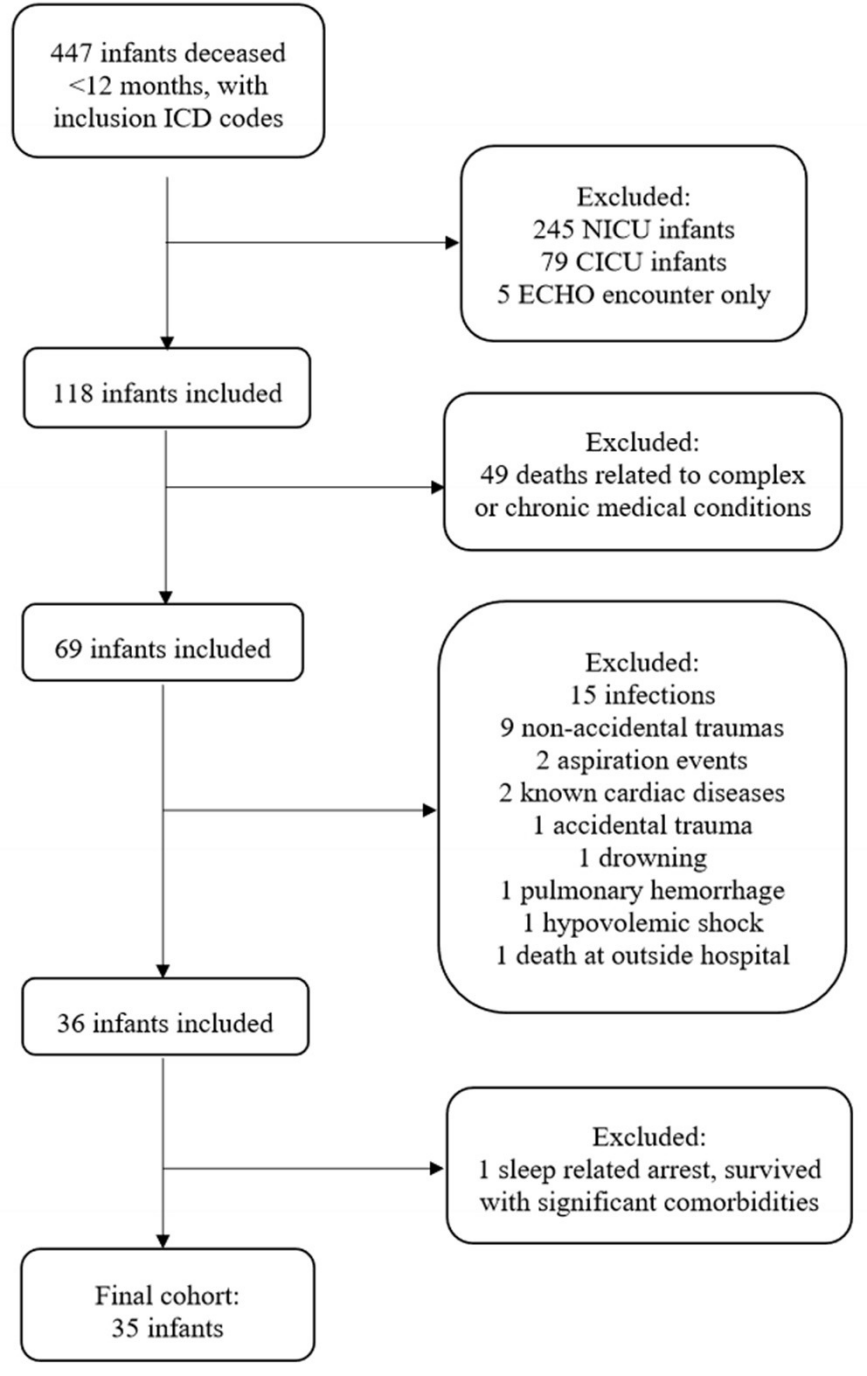
Flowchart of infant inclusion and exclusion, 2010–2020.

We performed a retrospective chart review of infants whose death was attributed to SUID over the last 10 years (December 2010 – December 2020) at a single tertiary care institution in the United States of America. The study population included patients aged 0–12 months who were declared deceased and assigned an ICD-9 or ICD-10 diagnosis of SIDS, SUID, death not otherwise specified (NOS), asphyxia, accidental suffocation and strangulation in bed (ASSB), suffocation, shaken infant syndrome, hypoxic ischemic encephalopathy, cardiac arrest, respiratory arrest, or unresponsive. Due to institutional admission practices, infants whose death occurred during a NICU or cardiac intensive care unit (CICU) encounter were excluded, leaving patients who presented to the emergency room (ER) or pediatric intensive care unit (PICU) (Figure 1). Infants whose cause of death was attributed to a known, underlying complex or chronic disease (e.g., genetic conditions, congenital syndromes, immunologic deficiencies, liver disease, etc.) were excluded. Deaths attributed to acute but identifiable non-sleep related causes (e.g., non-accidental trauma, sepsis, drowning, aspiration, etc.) were excluded. Finally, we excluded one infant who was identified as having a cardiac arrest attributed to unsafe sleep positioning and resultant suffocation, but was revived and survived to discharge.

A single investigator (KS) reviewed all patient charts that met inclusion criteria. Baseline descriptive characteristics, sleep patterns, and prior healthcare encounters were manually extracted from the electronic health record (EHR) for all patients who met inclusion criteria. Due to the retrospective nature of this study, data collection was reliant on manual review of clinical notes from the infant's final hospital encounter. Extraction of data (demographics, sleep patterns, healthcare encounters) was dependent on providers charting the presence or absence of clinical variables at the time of SUID. These variables were described in physician or social work notes and were based on parental report at the time of SUID event. Clinical notes reviewed for this study included: ER notes, history and physicals, progress notes, child protection team notes, social work notes, and death summaries.

Appropriate infant sleep patterns were defined as: in a crib/bassinette, supine position, absence of co-sleeping, and absence of pillows/blankets. Infants were determined to not meet AAP safe sleep guidelines if one or more of the above safe sleep definitions were not met. For demographics and sleep patterns, failure to document presence or absence of a variable was coded as “unknown.” Prenatal care was summarized as “adequate,” “delayed,” or “unknown” per physician or social worker documentation in their clinical note(s). Prior healthcare encounter was defined as any visit with a physician, nurse practitioner, or physician's assistant for a sick or well visit after discharge from the newborn nursery. Outpatient visits with the primary care provider (PCP) and urgent care were included. Emergency room visits, admissions to the general pediatric inpatient ward, admissions to the NICU, and admissions to the PICU were included. Only healthcare encounters that occurred in our EHR or were documented as having occurred in our clinical notes were included. We did not have access to encounters related to prenatal care, newborn nursery visits, or immunization-only visits, except as were documented in clinical notes at the time of SUID event as having previously occurred. For healthcare encounters, absence of documentation of previous exposure was coded as “no known exposure.”

## Results

### Demographics

A total of 35 infants met inclusion criteria, with a median age of 67 days (interquartile range [IQR] 48–112). There was a median of two adults in the home (IQR 2–3) and two minors in the home (IQR 1–3). Forty-three percent of caregivers reported receiving adequate prenatal care, while caregiver prenatal care status was unknown for 52% of the cohort. The majority (63%) of patients were fully immunized and 29% of patients were born premature at <37 weeks gestation. Although infrequent, known exposures to smoke (14%), alcohol (9%), and drugs (6%) were reported. Finally, 20% of our cohort had prior Department for Children and Family Services (DCFS) involvement, with 11% of families experiencing a prior child death (Table 1). DCSF cases involved prior fractures (*n* = 2), inadequate supervision (*n* = 2), death of previous infant (*n* = 2), and drug exposure at birth (n=1). Prior child death was attributed to SUID event while sleeping (*n* = 2), gas leak (*n* = 1), and gang violence (*n* = 1). Of note, one of these prior child deaths was an infant already in our cohort, as one family had two infants die from separate sleep-related events during the study period.

**Table 1:** Demographics of infants with SUID from 2010–2020 (*n* = 35).

	**Number (%)**
**Gender**	
Female	10 (28.6%)
Male	25 (71.4%)
**Age at SUID event**	
0–30 days	5 (14.3%)
30–60 days	9 (25.7%)
60–90 days	10 (28.6%)
90–120 days	6 (17.1%)
>120 days	5 (14.3%)
**Race and Ethnicity**	
African American	18 (51.4%)
Asian	1 (2.9%)
Caucasian	11 (31.4%)
Hispanic	4 (11.4%)
Other	1 (2.9%)
**Gestation (weeks)**	
Term (>37)	22 (62.9%)
Late preterm (34–36.6)	5 (14.3%)
Moderate preterm (32–33.6)	3 (8.6%)
Very preterm (28–31.6)	1 (2.9%)
Extreme preterm (<28)	1 (2.9%)
Unknown	3 (8.6%)
**Multiples**	
Twin	5 (14.3%)
Singleton/ unknown	30 (85.7%)
**Vaccine status**	
Fully immunized	22 (62.9%)
Partially immunized	1 (2.9%)
Unimmunized	2 (5.7%)
Unknown	10 (28.6%)
**Prenatal care**	
Adequate prenatal care	15 (42.9%)
Delayed prenatal care	2 (5.7%)
Unknown	18 (51.4%)
**Exposures**	
Known smoke exposure	5 (14.3%)
Known alcohol exposure	3 (8.6%)
Known drug exposure	2 (5.7%)
**Other risk factors**	
DCFS involved previously	7 (20%)
Prior child death	4 (11.4%)

*SUID, sudden unexpected infant death; DCFS, department of children and family services*.

### Sleep Patterns

Thirty-one percent of families routinely met the ABCs of safe sleep, however only 23% were in a safe sleep environment at the time of SUID (Table 2). All unsafe sleep behaviors were increased at the time of SUID compared to routine sleep patterns, with the highest incidences being inappropriate location (60% during the event vs. 20% during routine practice), co-sleeping (46% during the event vs. 23% during routine practice), and inappropriate position (37% during the event vs. 6% during routine practice) (Table 2).

**Table 2:** Overview of the infant sleep environment (*n* = 35).

	**Routinely**	**At time of SUID**
	***n*** **(%)**	***n*** **(%)**
**Overall safe sleep**		
Yes	11 (31.4%)	8 (22.9%)
No	8 (22.9%)	22 (62.9%)
Unknown	16 (45.7%)	5 (14.3%)
**Location**		
Crib/bassinette	11 (31.4%)	9 (25.7%)
Adult bed	6 (17.1%)	12 (34.3%)
Couch	1 (2.9%)	4 (11.4%)
Car seat	0 (0.0%)	1 (2.9%)
Held	0 (0.0%)	3 (8.6%)
Rock ‘n play	0 (0.0%)	1 (2.9%)
Unknown	17 (48.6%)	5 (14.3%)
**Position**		
Supine	3 (8.6%)	7 (20%)
Prone	2 (5.7%)	6 (17.1%)
Side	0 (0.0%)	3 (8.6%)
Held	0 (0.0%	4 (11.4%)
Unknown	30 (85.7%)	15 (42.9%)
**Sleep details**		
Co-sleeping	8 (22.9%)	16 (45.7%)
Presence of pillows/blankets	3 (8.6%)	7 (20%)

*SUID, sudden unexpected infant death*.

### Healthcare Encounters

Our cohort had 54 documented healthcare encounters (mean of 1.5 encounters per patient ± 2.1) prior to SUID (Table 3). Encounters with the primary care physician (57%) and NICU (29%) were the most frequent prior healthcare encounters, however visits spanned multiple specialties (Table 3). Of those who were admitted to the NICU previously, average length of stay (LOS) was 19 days (IQR 11–39). Inpatient general pediatric admissions had an average LOS of 2 days (IQR 2–2.5) and the single patient with prior PICU admission had a LOS of 19 days. Notably, 26% of our cohort had a documented healthcare encounter within 7 days of their death; the majority of these infants (78%) were seen by their primary care physician during this period.

**Table 3:** Healthcare encounters prior to SUID.

	**Total # visits**	**Average # visits**	**Exposure anytime**	**Exposure within 7 days**	**Median LOS in**
		**per patient**	**(*n* = 35)**	**of death (*n* = 35)**	**days (IQR)**
Any encounter^a^	54	1.5 ± 2.1	26 (74.3%)	9 (25.7%)	
PCP	36	1.0 ± 1.7	20 (57.1%)	7 (20%)	
ER	4	0.1 ± 0.4	3 (8.6%)	1 (2.9%)	
NICU	10	0.3 ± 0.5	10 (28.6%)	1 (2.9%)	19 (11, 39.3)
Inpatient pediatrics	3	0.1 ± 0.3	3 (8.6%)	1 (2.9%)	2 (2, 2.5)
PICU	1	0 ± 0.2	1 (2.9%)	1 (2.9%)	19

a *Includes PICU, NICU, Inpatient pediatrics, ER, and PCP encounters; does not include prenatal visits, immunizations-only visits, newborn nursery care, or subspecialty outpatient care encounters*.

*SUID, sudden unexpected infant death; PCP, primary care physician; ER, emergency room; NICU, neonatal intensive care unit; PICU, pediatric intensive care unit; LOS, length of stay*.

## Discussion

Despite AAP recommendations for infant safe sleep practices, families frequently practice unsafe sleep behaviors, with studies showing only 80% of families routinely place their infant supine, 60% practice room-sharing without bed-sharing, 40% avoid soft bedding, and 30% use a separate bed surface (14). Rates of unsafe sleep practices in SUID are further increased relative to the general population, supporting the relationship between SUID and unsafe sleep (15, 16). Our study supports this association, with similar unsafe sleep practices described during routine sleep in our cohort compared to general Unites States population data (14), and further increased at the time of SUID (15–17). With a well-documented relationship between SUID and unsafe infant sleep, promoting infant safe sleep practices is essential.

Several studies have examined infant safe sleep counseling by healthcare professionals, with overall trends not adhering to AAP recommendations. In the first decade after the Back to Sleep guidelines were published (18), 50–80% of physicians caring for pregnant women and infants routinely discussed SIDS, with only 40% recommending exclusively placing an infant supine to sleep (19). More recent survey data shows that 20% of parents report not receiving advice from doctors on infant sleep position, and over 50% report not receiving advice on sleep location (20). Furthermore, parental report of physician advice was frequently inconsistent with AAP guidelines, including >25% of parents reporting inconsistent recommendations for sleep position or location (20). Importantly, healthcare provider safe sleep advice is associated with increased infant safe sleep practices (14). While knowledge acquisition has been the foundation for the majority of prior safe sleep educational initiatives, recent literature suggests that future efforts would benefit from more targeted and tailored approaches that emphasize understanding, trust, and credibility (21). Ongoing efforts should address previously identified barriers based in culture, tradition, family support, available resources, and concerns for infant comfort and safety (22).

Historically, SUID and safe sleep educational efforts were focused on the NICU and newborn nursery encounters (7). Our results suggest a need to re-balance these efforts to include outpatient primary care practices (the most frequent presentation in our cohort) and inpatient hospitalizations (where length of stay provides multiple opportunities for education and modeling). By describing the frequency and locations of healthcare encounters prior to SUID, our study builds on existing literature to emphasize the importance of safe sleep counseling at every opportunity. Increased education for physicians on existing AAP safe sleep guidelines, including education on modeling and delivery of safe sleep advice, may increase safe sleep practices at home. Additionally, our findings highlight the breadth of healthcare professionals (e.g., neonatologists, hospitalists, outpatient providers, and emergency medicine physicians) who interact with families prior to SUID. All of these healthcare professionals have the opportunity to educate families about SUID and potentially improve safe sleep practices in the home.

Our result of 1.5 mean healthcare encounters per patient is likely a gross underestimate. While our study comments on the presence or absence of prenatal care (if known) prior to SUID, we did not include prenatal encounters when calculating average number of visits per patient. Prior studies describe an average of 8–12 prenatal care visits per mother, making this encounter type a recurrent interaction for families and another potential target for interventions (23). Additionally, newborn nursery visits (spanning 24–72h after birth for most infants) were not included in our results due to the inability to reliably extract this data. On our medical campus, the birthing center is part of a separate Women's Hospital, and therefore nursery visit encounters were unavailable for review. Prenatal care and nursery visits are major opportunities for parental safe sleep education due to the frequency of visits and duration of newborn stay.

Other limitations worth noting are that our study was conducted at a single academic center, therefore limiting our sample size. Due to the retrospective nature of this study, data extraction was reliant on clinical notes documenting safe sleep and demographic information at the time of SUID. In many patient charts, the data was incompletely documented, resulting in missing variables. Institutional quality improvement efforts should include development of a standardized and comprehensive data collection tool to use following a suspected sleep-related death. Additionally, the nature of using a single institution's EHR limited the total healthcare encounter estimates as we were unable to view encounters from healthcare facilities outside our network. Next, variability in ICD code designation at the time of death may have resulted in missing SUID patients. Autopsies primarily occurred at the county level and results were unavailable for review. Therefore, cause of death, if identified on autopsy, was not available to report.

This topic would benefit from a larger scale, multi-center study to more thoroughly describe healthcare interactions prior to SUID. By better describing where families present in the healthcare system prior to their child's death, we can better target educational efforts for healthcare providers, improve advice for families, and direct future policy change. Additionally, future quality improvement efforts would benefit from investigation of family attitudes toward safe sleep/SUID education provided by healthcare professionals at various encounters in the first year of life. With increased understanding of family attitudes surrounding healthcare provider education, we can deepen our communication and build trust with families. Heightened awareness and knowledge of the SUID cohort may result in improved emphasis on safe sleep education and modeling by healthcare professionals across specialties. Hopefully, one day this translates into optimized safe sleep practices for families at home and reduced incidence of SUID.

## Conclusion

We created a local database of infants who died from SUID, demonstrating the frequency and variability in healthcare encounters among these infants prior to death. The majority of infants had prior healthcare encounters, with 26% of our cohort seen by healthcare professionals within 7 days of their death. These results highlight the important role healthcare professionals across all specialties have the potential to play in educating families about safe sleep and SUID risk factors. Increased emphasis on this education during all infant encounters may be an opportunity to reduce the risk of SUID.

## Data Availability Statement

The raw data supporting the conclusions of this article will be made available by the authors, without undue reservation.

## Ethics Statement

Ethical review and approval was not required for the study on human participants in accordance with the local legislation and institutional requirements. Written informed consent from the participants' legal guardian/next of kin was not required to participate in this study in accordance with the national legislation and the institutional requirements.

## Author Contributions

KS conceptualized and designed the study, collected the data, carried out the initial analysis and interpretation of data, drafted the initial manuscript, and reviewed and revised the manuscript. CB conceptualized and designed the study, carried out the initial analysis and interpretation of data, and reviewed and revised the manuscript for important intellectual content. Both authors approved the final manuscript as submitted and agree to be accountable for all aspects of the work.

## Conflict of Interest

The authors declare that the research was conducted in the absence of any commercial or financial relationships that could be construed as a potential conflict of interest.

## Publisher's Note

All claims expressed in this article are solely those of the authors and do not necessarily represent those of their affiliated organizations, or those of the publisher, the editors and the reviewers. Any product that may be evaluated in this article, or claim that may be made by its manufacturer, is not guaranteed or endorsed by the publisher.

## Abbreviations

SIDS: sudden infant death syndrome
SUID: sudden unexpected infant death
ABCs, Alone, on the Back: in an empty Crib
NICU: neonatal intensive care unit
AAP: American academy of pediatrics
NOS: not otherwise specified
ASSB: accidental suffocation and strangulation in bed
CICU: cardiac intensive care unit
ER: emergency room
PICU: pediatric intensive care unit
EHR: electronic health record
PCP: primary care physician
DCFS: department of children and family services
LOS: length of stay.
